# The Function of Oncogene B-Cell Lymphoma 6 in the Regulation of the Migration and Invasion of Trophoblastic Cells

**DOI:** 10.3390/ijms21218393

**Published:** 2020-11-09

**Authors:** Andreas Ritter, Babek Khan Safdar, Britta Jasmer, Nina-Naomi Kreis, Alexandra Friemel, Susanne Roth, Christine Solbach, Frank Louwen, Juping Yuan

**Affiliations:** Obstetrics and Prenatal Medicine, Department of Gynecology and Obstetrics, University Hospital, J. W. Goethe-University, Theodor-Stern-Kai 7, D-60590 Frankfurt, Germany; Babek.khan@gmail.com (B.K.S.); britta_ja@gmx.de (B.J.); Nina-Naomi.Kreis@kgu.de (N.-N.K.); Alexandra.Friemel@kgu.de (A.F.); Susanne.Roth@kgu.de (S.R.); Christine.Solbach@kgu.de (C.S.); Louwen@em.uni-frankfurt.de (F.L.)

**Keywords:** BCL6, trophoblast, cell and focal adhesion, actin dynamics, migration and invasion

## Abstract

Human placentation is a highly invasive process. Deficiency in the invasiveness of trophoblasts is associated with a spectrum of gestational diseases, such as preeclampsia (PE). The oncogene B-cell lymphoma 6 (BCL6) is involved in the migration and invasion of various malignant cells. Intriguingly, its expression is deregulated in preeclamptic placentas. We have reported that BCL6 is required for the proliferation, survival, fusion, and syncytialization of trophoblasts. In the present work, we show that the inhibition of BCL6, either by its gene silencing or by using specific small molecule inhibitors, impairs the migration and invasion of trophoblastic cells, by reducing cell adhesion and compromising the dynamics of the actin cytoskeleton. Moreover, the suppression of BCL6 weakens the signals of the phosphorylated focal adhesion kinase, Akt/protein kinase B, and extracellular regulated kinase 1/2, accompanied by more stationary, but less migratory, cells. Interestingly, transcriptomic analyses reveal that a small interfering RNA-induced reduction of BCL6 decreases the levels of numerous genes, such as p21 activated kinase 1, myosin light chain kinase, and gamma actin related to cell adhesion, actin dynamics, and cell migration. These data suggest BCL6 as a crucial player in the migration and invasion of trophoblasts in the early stages of placental development through the regulation of various genes associated with the migratory machinery.

## 1. Introduction

The oncogene B-cell lymphoma 6 (BCL6), which is a crucial regulator of B-lymphocyte development, is a transcriptional modulator and often deregulated in lymphomas [1,2]. Along with B-cell activation and differentiation, BCL6 plays roles in the DNA damage response, cell cycle regulation, and apoptosis induction of lymphocytes [3,4], as well as in the invasion and migration of breast cancer cells [5]. Moreover, in ovarian and breast tissues, deregulated BCL6 blocks the differentiation of epithelial cells, supporting malignant progression [2,6,7].

Interestingly, the gene expression of BCL6 has been reported to be increased in preeclamptic placentas [8,9,10,11,12]. We have recently shown an increase of BCL6 in preeclamptic placentas at an mRNA and protein level [13]. BCL6 promotes proliferation [14] and affects the fusion of villous trophoblastic cells (vCTBs) [15]. In term placentas, BCL6 is predominantly found in vCTBs directly underneath the syncytiotrophoblast layer and only weakly expressed in extravillous trophoblasts (EVTs) [13,14,15], suggestive of being less significant in term EVTs. In contrast, BCL6 is highly expressed in EVT cell line HTR-8/SVneo derived from a first trimester placenta, indicative of its involvement in the EVT function of early stages of the placenta.

A healthy placenta is the key to a successful pregnancy and the life-long health of the offspring [16] and human placentation is a highly invasive process [17], which shares various characteristics with cancer cell invasion [18]. At the feto–maternal interface, villous cytotrophoblast cells differentiate into syncytiotrophoblasts (STBs) or extravillous trophoblasts. EVTs migrate to the maternal side for implantation or infiltrate the uterine spiral arterioles, remodeling the endothelium lining of the vessels [19,20]. As a result, the vessels dilate to ensure a constant high volume and low velocity of maternal blood flow to the placenta [21]. The migration and invasion of EVTs are thus fundamental for proper placentation and sufficient arterial remodeling. Deficiencies in these processes have been associated with a spectrum of gestational diseases, such as preeclampsia (PE) [22,23]. These processes require immune tolerance modulation and a distinctive invasive capacity for EVTs, which are also required in malignant cells for tumor progression [18].

PE, which is the most common disease in pregnancy, is associated with substantial maternal and fetal morbidity and mortality worldwide [24,25]. It affects 4–8% of pregnancies, and is associated with 12% of infants with fetal growth restriction and approximately 20% of preterm deliveries [25,26]. PE is defined as the presence of new onset hypertension and proteinuria or other end-organ damage occurring after 20 weeks of gestation [27]. The most important observations in its pathogenesis are inadequate trophoblast invasion and improper spiral artery remodeling. Consequently, these events trigger a restriction in uteroplacental circulation and result in an insufficiently perfused, ischemic, and hypoxic placenta. PE, which is a clinical syndrome that has been known for centuries, remains incurable and early termination of pregnancy is the only definitive treatment [28].

We hypothesized about the role of BCL6 in the motility of trophoblasts in early placental development. In the present study, we show that BCL6 is involved in the regulation of migration and invasion on multiple levels of trophoblastic cells derived from first trimester placental tissue. Moreover, a comparison to previous transcriptome data of BCL6 in epidermoid carcinoma cells reveals that BCL6 targets the same genes in trophoblastic cells and malignant cells, likely to modulate cell invasion.

## 2. Results

### 2.1. Abundance of BCL6 in First Trimester Placentas Required for the Migration of Trophoblastic Cells

**Figure 1:**
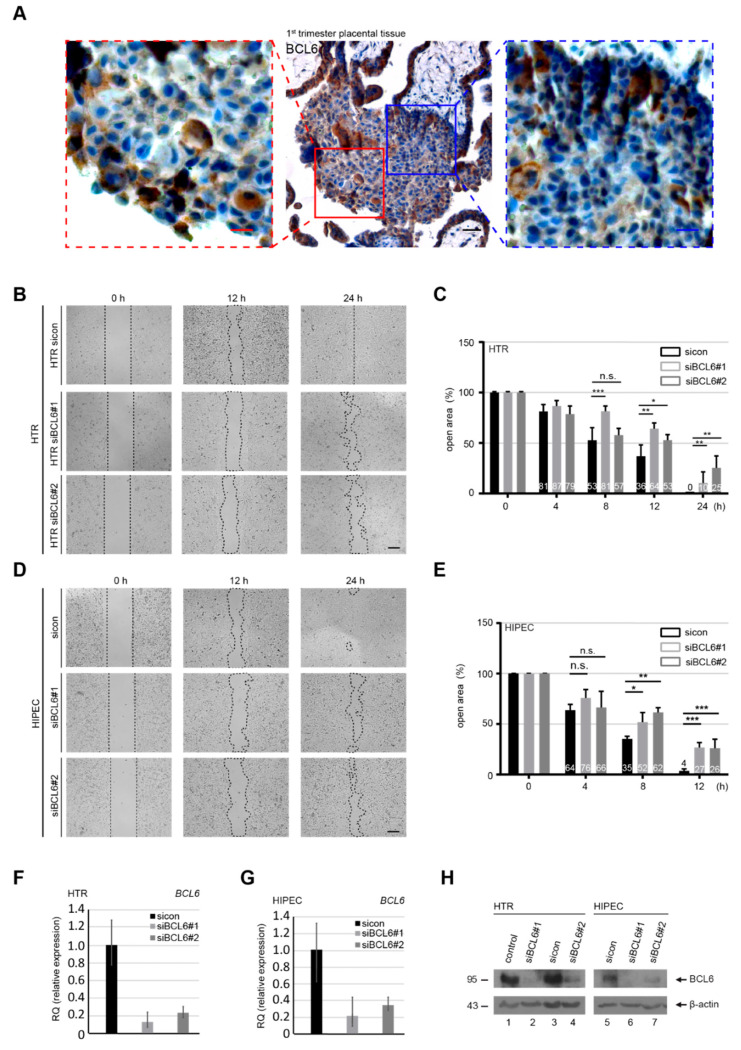
Abundance of B-cell lymphoma 6 (BCL6) in first trimester placentas and requirement for the migration of trophoblastic cells. (**A**) First trimester placental tissue sections were immunohistochemically stained for BCL6 and DNA. Representatives are shown. Scale: 50 μm, inset scale: 20 μm. (**B**,**D**) HTR-8/SVneo (HTR) (**B**) or HIPEC-65 (HIPEC) (**D**) cells were treated with control siRNA (sicon) or two different siRNAs targeting the coding region of BCL6 (siBCL6#1 and siBCL6#2) for 12 h. Treated cells were subjected to wound healing/migration assays. Migration front closure was imaged at 0, 8, and 12 h and bright-field images are shown. The migration front is visualized by black dashed lines. Scale: 300 μm. (**C**,**E**) The open areas of HTR (**C**) or HIPEC (**E**) cells were quantified and are presented in a bar graph at multiple time points. The reference point was the cell-free area at 0 h, which was set as 100%. Data were obtained from three independent experiments and are shown as the mean ± SEM. *** *p* < 0.001, ** *p* < 0.01, * *p* < 0.05 and, n.s. *p* > 0.05. (**F**–**H**) Relative BCL6 gene levels in treated HTR (**F**) and HIPEC (**G**) cells were measured as transfection efficiency controls, as well as the protein levels analyzed by Western blot.

To examine whether BCL6 is expressed in early EVTs, placental tissue sections from first trimester placentas were immunohistochemically stained for BCL6. As shown in Figure 1A, BCL6 is richly expressed in EVTs migrating from anchoring villi to proximal cell columns, suggestive of its role in early EVTs of the placenta. Additionally, BCL6 is also expressed in STBs and vCTBs in first trimester placentas, as can be seen in Figure 1A. To address whether BCL6 is involved in migration, HTR-8/SVneo (referred to hereafter as HTR), which is a well-established first trimester trophoblast cell line [29], was treated with control siRNA (sicon), and siRNA No. 1 and No. 2 specifically targeting the coding region of BCL6 (siBCL6#1 and siBCL6#2) for the wound-healing/migration assay. HTR cells were efficiently depleted of BCL6 on the gene level (Figure 1F), which was also true for the protein level (Figure 1H, lane 1–4). Compared to sicon-treated cells, HTR cells treated with siBCL6#1 and siBCL6#2 exhibited a compromised migratory capability of 44% and 33%, respectively, at 12 h (Figure 1B,C). At 24 h, control HTR cells closed the open area, whereas BCL6-depleted HTR cells still exhibited 10–25% open areas (Figure 1B,C). To corroborate these results, migration assays were carried out with HIPEC-65 cells (referred to hereafter as HIPEC), representing another well-characterized first trimester trophoblast cell line [30]. In line with the results from HTR cells, the migration of HIPEC cells was also impaired (Figure 1D,E) upon the depletion of BCL6 (Figure 1G,H, lane 5–7). These observations were further underscored with HTR cells treated with 79-6 (Figure S1A), which is a small molecule inhibitor targeting BCL6 [31]. Moreover, while 79-6 showed a slight inhibitory effect at 100 µM (Figure S1B), the knockdown of BCL6 also impaired the migration of BeWo cells (Figure S1C–E), representing a trophoblastic cell line derived from choriocarcinoma [32]. These findings suggest that BCL6 is required for the migration of EVTs from first trimester placentas, as well as malignant trophoblastic BeWo cells.

### 2.2. Suppression of BCL6 Reduces the Motility and Invasiveness of EVT Cells

**Figure 2:**
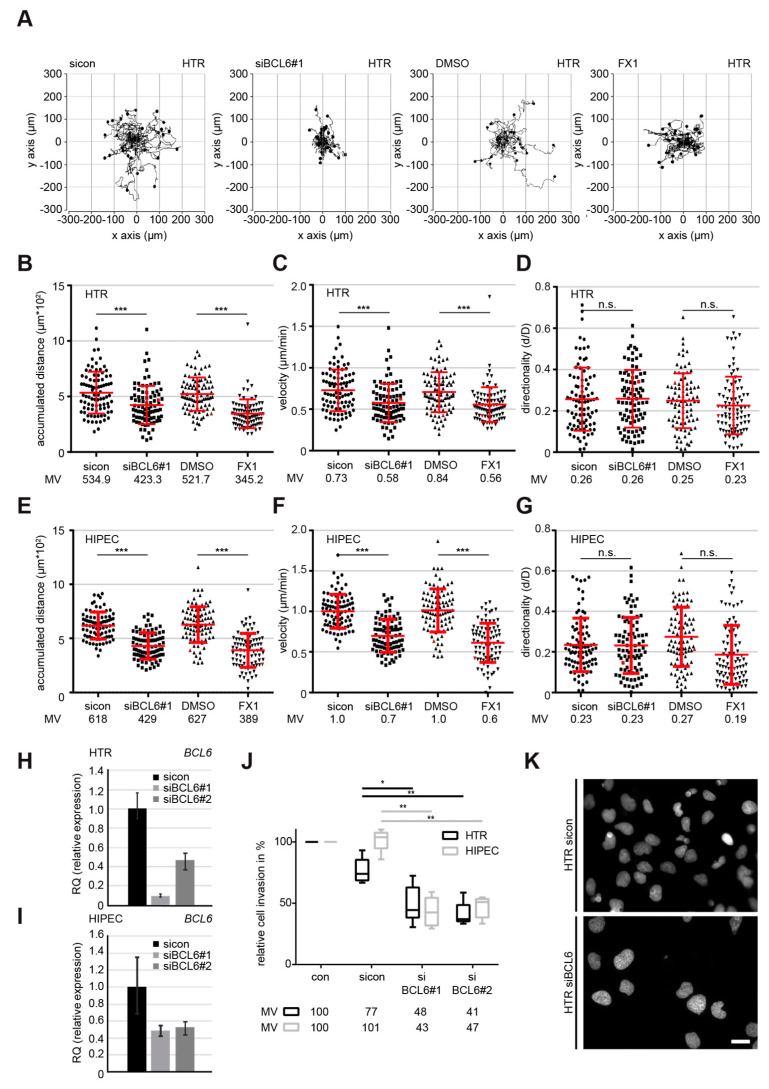
Inhibition of BCL6 reduces the motility of first trimester trophoblastic cells. (**A**–**D**) Time-lapse microscopy was performed with HTR cells, depleted of BCL6 with siBCL6#1 or treated with the BCL6 inhibitor FX1 for up to 12 h. The random motility of these cells was analyzed. Representative trajectories of individual cells (*n* = 30) are shown (**A**). The evaluated accumulated distance (**B**), velocity (**C**), and directionality (**D**) from three independent experiments are presented as scatter plots with variations. *** *p* < 0.001, * *p* < 0.05 and, n.s. *p* > 0.05. (**E**–**G**) Time-lapse microscopy was performed with HIPEC cells, depleted of BCL6 with siBCL6#1 or treated with the BCL6 inhibitor FX1 for up to 12 h. The random motility of these cells was analyzed. The evaluated accumulated distance (**E**), velocity (**F**), and directionality (**G**) from three independent experiments are shown as scatter plots with variations. *** *p* < 0.001. (**H**–**K**) Invasion assay. HTR or HIPEC cells were treated with sicon or siBCL6#1, seeded into Transwell systems, and starved for 12 h. The cells were released into fresh medium for 24 h and fixed for quantification. The BCL6 gene levels were measured in HTR (**H**) or HIPEC cells (**I**) as transfection efficiency controls. Representatives of invaded HTR cells are shown (**K**). Scale: 25 μm. Invaded cells were quantified. The total number of invaded cells in the control group was assigned as 100%. The results were obtained from three independent experiments and are presented as the mean ± SEM (**J**). ** *p* < 0.01.

To underline the observation, individual cell tracking using time-lapse microscopy, which is a well-used assay employed to document the motility of single cells [33,34,35], was performed with HTR cells. Single interphase cells treated with sicon, siBCL6#1 (Figure 2H), vehicle control DMSO, or FX1, the latter of which is a specific small molecule BCL6 inhibitor [36], were tracked for 12 h, as shown with representative trajectories of individual cells (Figure 2A). Evaluation analysis revealed that both the accumulated distance and the velocity were significantly decreased in HTR cells after the suppression of BCL6 via siRNA#1 or the small molecule inhibitor FX1 compared to control cells (Figure 2B,C), whereas there was hardly any alteration in directionality (Figure 2D). In comparison to control cells, the depletion and inhibition of BCL6 reduced the accumulated distance by 20% and 34% (Figure 2B), respectively. In parallel, the velocity was decreased to 80% in HTR cells depleted of BCL6 and 66% in HTR cells treated with FX1 (Figure 2C). Comparable results were also obtained with HIPEC cells (Figure 2E–G,I, and Figure S2A), as well as with SGHPL-4 cells (Figure S2B–F), which is a third widely-used EVT cell line derived from a first trimester placenta [37]. In addition, the exogenous expression of BCL6 slightly increased the accumulated distance and the velocity of HTR cells, with hardly any alteration in directionality (Figure S2G–K).

Moreover, HTR and HIPEC cells knocked down of BCL6 (Figure 2H,I) were subjected to an invasion assay. Relative to control cells, only 48% of siBCL6#1-treated and 41% of siBCL6#2-treated HTR cells were able to invade through the Matrigel layer (Figure 2J,K). Similar results were also obtained after the treatment with siBCL6#1 or siBCL6#2, with 43% and 47% of HIPEC cells passing the layer, respectively (Figure 2I,J). Altogether, these data indicate that BCL6 is involved in the migration, motility, and invasion of EVTs derived from first trimester placentas.

### 2.3. BCL6 Depletion Reduces Cell Adhesion and Increases Filamentous Actin in HTR Cells

**Figure 3:**
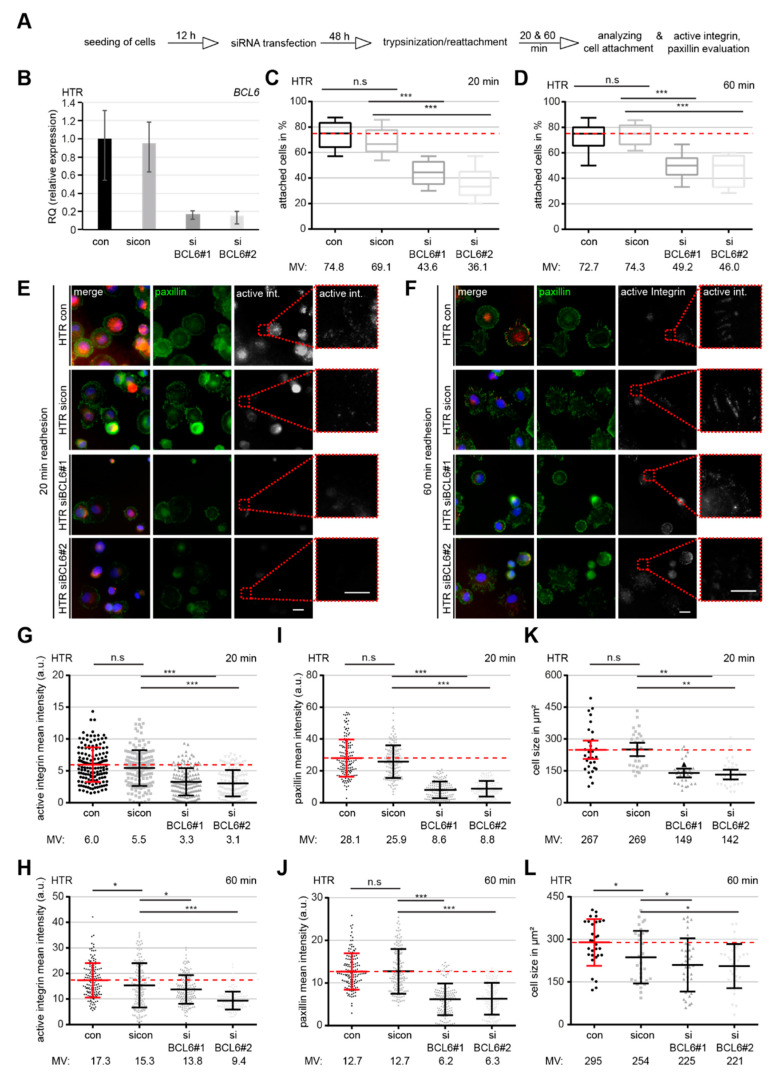
Cell adhesion and integrin activation are compromised upon BCL6 depletion. (**A**) Schedule of the cell spreading/adhesion assay. HTR cells were treated with sicon, siBCL6#1, or siBCL6#2. Forty-eight hours later, treated cells were trypsinized and reseeded for 20 or 60 min for a further evaluation of attached cells or for immunofluorescence staining. (**B**) The BCL6 gene levels were measured in treated cells as transfection efficiency controls. (**C**) Evaluation of attached cells at 20 min after reseeding (300 cells). The results are based on three independent experiments and presented as box plots. *** *p* < 0.001. (**D**) Quantification of attached cells at 60 min after reseeding. The results were obtained from three independent experiments and are shown as box plots, with bars indicating variable sizes (300 cells). *** *p* < 0.001. (**E**,**F**) Attached cells at 20 min (**E**) and 60 min (**F**) after reseeding were also stained for focal adhesion (FA) markers paxillin and active integrin, as well as DNA, for evaluation. Scale: 25 μm, inset scale: 20 μm. (**G**,**H**) Relative intensity of paxillin was evaluated at 20 min (**G**) and 60 min (**H**) after reseeding (280 FAs for each condition). The data were obtained from three independent experiments and are presented as scatter plots with variations. The red dashed line depicts the relative intensity measured in control cells. (**I**,**J**) Relative intensity of active integrin was evaluated at 20 min (**I**) and 60 min (**J**) after reseeding (280 FAs for each condition). The results were derived from three independent experiments and are presented as scatter plots with variations. The red dashed line depicts the intensity measured in control cells. (**K**,**L**) The relative cell size was evaluated at 20 min (**K**) and 60 min (**L**) after reseeding (30 cells for each condition). The data were obtained from three independent experiments and are shown as scatter plots with variations. The red dashed line depicts the relative cell size measured in control cells. For **G**–**L**: *** *p* < 0.001, ** *p* < 0.01, * *p* < 0.05 and, n.s. *p* > 0.05.

The interplay of cell adhesion and actin dynamics is crucial for migration [38]. To address whether BCL6 affects the behavior of cell adhesion, HTR cells knocked down of BCL6 were trypsinized and reseeded for 20 or 60 min to analyze the percentage of attached cells (Figure 3A). siBCL6#1 and siBCL6#2 worked efficiently in HTR cells (Figure 3B). At 20 min post reseeding, HTR cells treated with siBCL6#1 or siBCL6#2 exhibited less reattachment ability, with values of 63% and 52%, respectively, relative to sicon-treated cells (Figure 3C). At 60 min post reseeding, only a slight improvement was achieved, showing that 66% of siBCL6#1-treated and 62% of siBCL6#2-treated HTR cells were attached (Figure 3D). The same experiment was also carried out with HIPEC cells and similar findings were observed (Figure S3A–C). These data suggest a role of BCL6 in the regulation of cell adhesion, either directly or indirectly.

Dynamic focal adhesion (FA) is crucial for the regulation of cell attachment [39]. To look at the potential involvement of BCL6 in FA dynamics, siRNA-treated HTR cells were stained for adhesion markers active integrin and paxillin (Figure 3E,F). The quantitative analysis revealed that the relative intensity of active integrin was reduced upon the depletion of BCL6 with siBCL6#1 or siBCL6#2 at 20 min, as well as at 60 min, after reseeding (Figure 3G,H). As expected, the relative intensity of paxillin was also significantly decreased in treated HTR cells at both time points (Figure 3I,J). In parallel, the cell size was smaller in HTR cells depleted of BCL6 than control cells (Figure 3K,L), indicating that cell attachment was not properly accomplished in HTR cells deficient of BCL6. The same experiments were also performed with HIPEC cells and comparable results were observed (Figure S3D–I). In sum, these results point to the notion that the assembly of FAs and the process of cell adhesion are not efficient in HTR and HIPEC cells with reduced BCL6.

**Figure 4:**
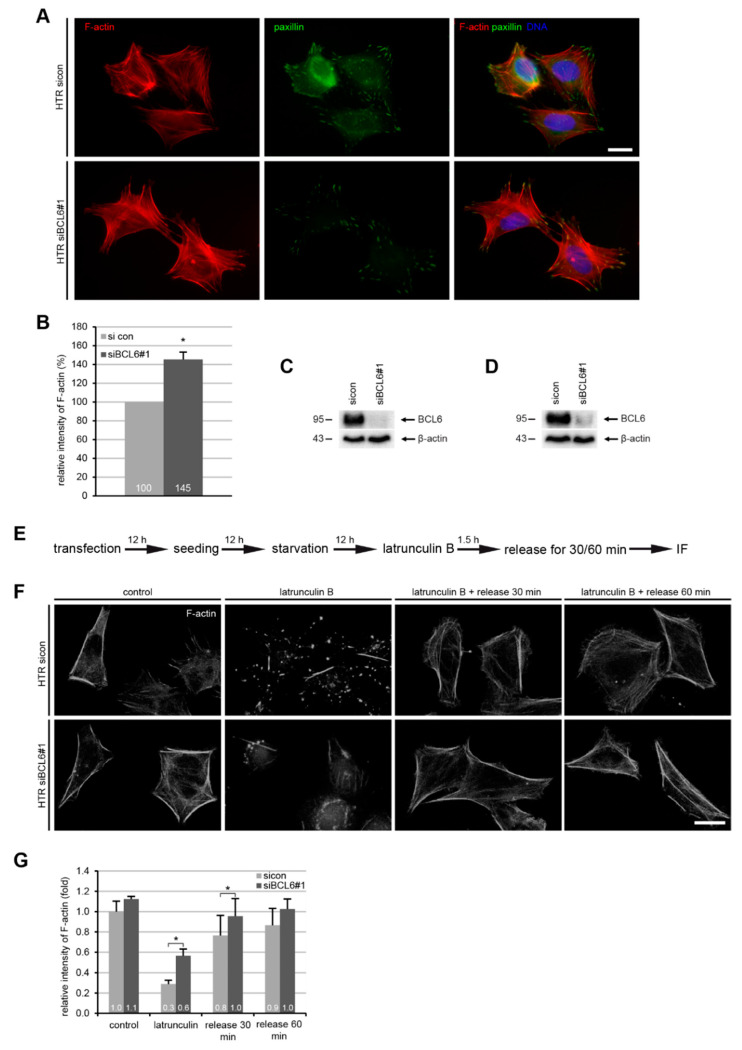
Elevated F-actin and reduced paxillin in HTR cells with reduced BCL6. (**A**) HTR cells, treated with control siRNA (sicon) or siRNA targeting BCL6 (siBCL6#1) for 24 h, were stained for F-actin and paxillin for microscopic evaluation. Representatives are shown. Scale: 25 μm. (**B**) The relative intensity of F-actin was measured in treated HTR cells (200 cells for each condition). The results were obtained from three independent experiments and are presented as the mean ± SEM. * *p* < 0.05. (**C**) Western blot analysis as the transfection efficiency control for (**A**,**B**). Β-actin served as the loading control. (**D**) Western blot analysis as the transfection efficiency control for (**F**,**G**). β-actin served as the loading control. (**E**) Schedule of the latrunculin B washout experiment. HTR cells, transfected with sicon or siRNA BCL6#1 for 24 h, where 10 µM latrunculin B was added into the medium for 90 min, and after 30 and 60 min, cells were released into fresh prewarmed culture medium. (**F**) Cells were stained for F-actin (phalloidin, white). Representatives images of the reassembly rate of actin fibers are presented. Scale: 25 µm. (**G**) The mean fluorescence intensity of F-actin (phalloidin) was quantified and is presented (30 cells were used per condition). The experiment was independent repeated three times and the results are presented as the mean ± SEM. * *p* < 0.05.

The actin cytoskeleton is the major source of internally generated force that regulates FA dynamics, cell shape, and migration [40]. To address this issue, HTR cells depleted of BCL6 (Figure 4C) were stained for filamentous actin (F-actin, phalloidin), FA marker paxillin, and DNA (Figure 4A) for microscopic evaluation. Compared to control cells, the relative intensity of F-actin was considerably enhanced in HTR cells treated with siBCL6#1 (Figure 4B), indicating an association between enhanced F-actin and reduced cell adhesion, as well as FA assembly. To study whether BCL6 affects the dynamics of actin polymerization, HTR cells deficient of BCL6 (Figure 4D) were subjected to the actin destabilizer latrunculin B, which is a reversible actin monomer-sequestering agent that blocks fast actin polymerization and abolishes stress fiber formation [41,42], for 90 min, and released for 30 and 60 min to re-polymerize actin (Figure 4E). Treated cells were stained for F-actin and its relative cellular amount was evaluated via a fluorescence intensity measurement. This analysis showed that there was 28% more F-actin left in HTR cells knocked down of BCL6 after latrunculin treatment, as well as after 20 and 60 min release (19%, and 16% increase with siBCL6#1, respectively) (Figure 4F,G), suggesting that BCL6 affects the turnover of filamentous actin and the dynamics of the actin cytoskeleton.

### 2.4. BCL6 Influences Multiple Signaling Pathways and Its Reduction is Associated with A Stationary Phenotype of HTR Cells

**Figure 5:**
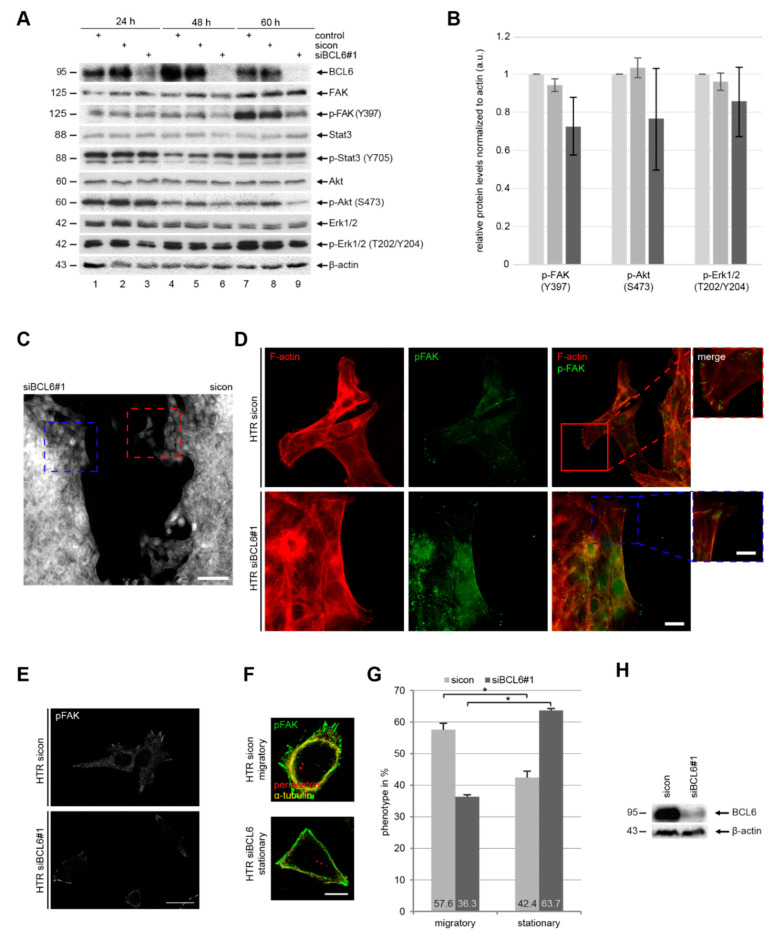
Deficiency of BCL6 weakens the activation of multiple signaling pathways. (**A**) HTR cells were treated with sicon or siBCL6#1 for indicated time points. Cellular extracts were prepared for Western blot analyzes with indicated antibodies. β-actin served as the loading control. (**B**) The relative ratios are depicted in this graph and the results are based on three independent experiments and presented as the mean ± SEM. (**C**,**H**) HTR cells depleted of BCL6 (**H**) and sicon cells were seeded for the wound healing/migration assay. Twelve hours later, cells were stained for F-actin (white). A representative overview of both migration fronts of two different cell conditions is shown. Scale: 300 µm. (**D**) Magnified representative images (sicon: red and siBCL6#1: blue) of the migration fronts are shown with indicated antibodies. Scale: 25 µm, inset scale: 12.5 µm. (**E**) HTR cells depleted of BCL6 (**A**) were also stained for *p*-FAK (white). Scale: 30 µm. Representative migratory and stationary HTR cells are shown (**F**). Scale: 25 µm. These two types of HTR cells were quantified (300 cells for each condition) and the results are based on three independent experiments and presented as the mean ± SEM (**G**). * *p* < 0.05.

BCL6, which is a transcriptional repressor, affects a great body of genes related to various signaling pathways [3,43]. To explore the molecular mechanisms behind these observations, HTR cells were treated with sicon or siBCL6#1 for 24, 48, and 60 h, respectively. Cellular lysates were prepared for Western blot analysis. HTR cells were efficiently depleted of BCL6 (Figure 5A, 1st row). Interestingly, the protein levels of focal adhesion kinase (FAK), which is an important regulator of cell adhesion and migration [39]; Akt/protein kinase B, which is the downstream target of the phosphoinositide 3-kinase (PI3K) pathway; and extracellular regulated kinase 1/2 (Erk1/2), which is the effector of the mitogen-activated protein kinase (MAPK), were scarcely altered (Figure 5A, 2nd, 6th, and 8th rows, respectively). In contrast, their active phosphorylated forms were strongly reduced (Figure 5A, 3rd, 7th, and 9th rows, respectively). Of note, there was almost no difference regarding the protein level and its active form of the signal transducer and activator of transcription 3 (STAT3), which is involved in Akt and Erk signaling, between control HTR cells and HTR cells deficient of BCL6 (Figure 5A, 4th and 5th rows, respectively). Therefore, BCL6 affects migration and invasion, possibly via several intracellular pathways, including FAK, PI3K/Akt, and MAPK/Erk signaling, which are well-known to be tightly involved in cell motility [39,44,45].

To further delineate this issue, HTR cells were treated with sicon or siBCL6#1 (Figure 5H), seeded onto two chambers (migration assay chambers), and cultured, in order to allow cells to migrate to each other. Twelve hours later, cells were fixed and stained for phosphorylated FAK (*p*-FAK) and F-actin (phalloidin). Microscopic examination revealed a clear difference between the two opposite fronts formed by HTR cells treated with sicon and by HTR cells treated with siBCL6#1 (Figure 5C). While the front of control HTR cells exhibited many cell migratory protrusions (Figure 5C, right side, red insert), the front of HTR cells knocked down of BCL6 was blunt and flat (Figure 5C, left side, blue insert). By examining these cells in more detail, we found that control HTR cells formed lamellipodia with specific localized *p*-FAK, whereas HTR cells depleted of BCL6 displayed less lamellipodia and *p*-FAK mainly located in the cytoplasm (Figure 5D, green *p*-FAK staining), suggesting that the depletion of BCL6 renders them non-migratory or rather stationary. To underline this observation, HTR cells depleted of BCL6 (Figure 5H) were stained for *p*-FAK (Figure 5E) and the populations of migratory and stationary cells (Figure 5E) were evaluated (Figure 5G). Interestingly, this analysis showed that a reduction in BCL6 clearly increased the cell population of stationary cells and decreased the population of migratory cells (Figure 5G). These data demonstrate that the suppression of BCL6 reduces *p*-FAK, *p*-Akt, and *p*-Erk1/2, accompanied by decreased migratory and increased stationary HTR cells.

### 2.5. Reduced p-Paxilin and p-FAK Upon the Depletion or Inhibition of BCL6

**Figure 6:**
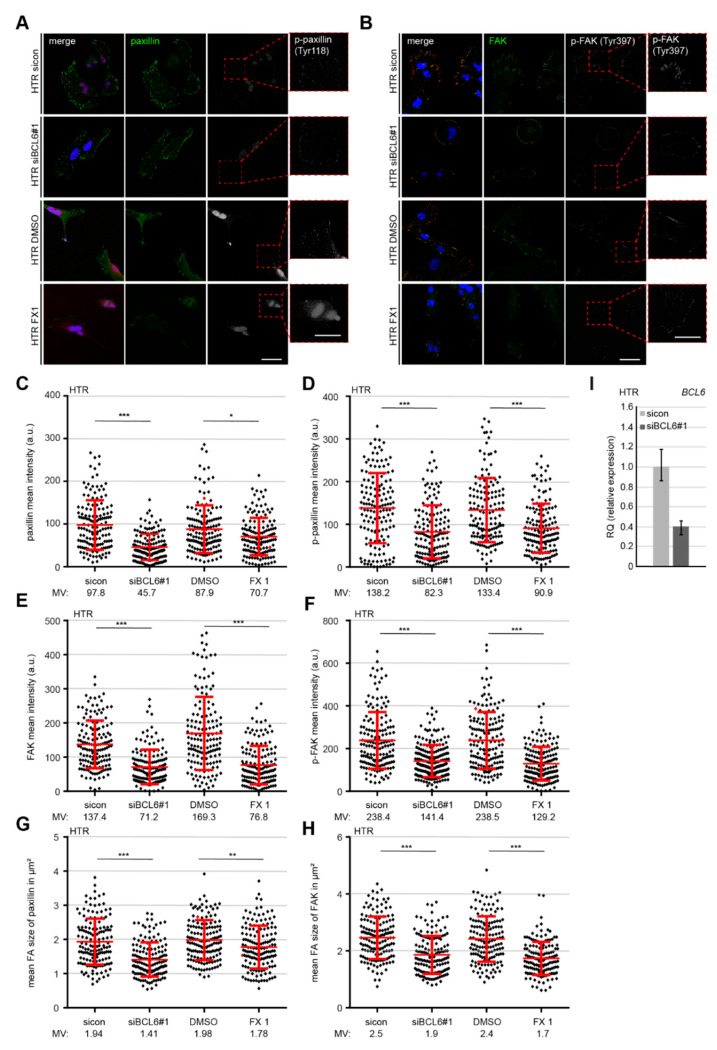
Reduced paxillin, *p*-paxillin, focal adhesion kinase (FAK), and phosphorylated FAK (*p*-FAK) at FAs of HTR cells deficient of BCL6. (**A**,**B**) HTR cells were treated with sicon or siBCL6#1, with DMSO or BCL6 inhibitor FX1. Twenty-four hours later, cells were stained for paxillin (green), *p*-paxillin (white), and DNA (blue, DAPI) (**A**), or for FAK (green), *p*-FAK (red), and DNA (blue, DAPI) (**B**). Representatives are shown. Scale: 25. Insets are magnified regions. Scale: 20. (**C**,**D**) Mean intensities of paxillin (**C**) and *p*-paxillin (**D**) were measured (280 FAs for each condition). The results were derived from three independent experiments and are presented as scatter plots with variations. a.u., arbitrary units. (**E**,**F**) Mean intensities of FAK (**E**) and *p*-FAK (**F**) were measured (280 FAs for each condition). The results were derived from three independent experiments and are presented as scatter plots with variations. a.u., arbitrary units. (**G**,**H**) Mean sizes of paxillin (**G**) or FAK (**H**) in FAs were measured (280 FAs for each condition). The results were obtained from three independent experiments and are presented as scatter plots with variations. (**I**) BCL6 gene analysis as the transfection efficiency control. For (**C**–**H**): *** *p* < 0.001, ** *p* < 0.01, and * *p* < 0.05.

Integrin clustering causes FAK autophosphorylation and affects cell motility [46]. Paxillin, which is an FA adaptor and scaffolding protein, is phosphorylated upon integrin clustering, partially by FAK [47]. To further characterize the crucial proteins in FAs, HTR cells, depleted of BCL6 or treated with the BCL6 inhibitor FX1, were stained for paxillin and its active form *p*-paxillin (S258) (Figure 6A), in addition to FAK and its active form *p*-FAK (Y397) (Figure 6B), for microscopic evaluation. Quantitative analysis showed a significant change in the intensity of paxillin and *p*-paxillin (Figure 6C,D), FAK and *p*-FAK (Figure 6E,F), and the mean FA size of stained paxillin and FAK of HTR cells (Figure 6G,H), upon the depletion (Figure 6I) or inhibition of BCL6. In particular, compared to control cells, the knockdown of BCL6 impaired the recruitment of paxillin by 54% (Figure 6C) and FAK by 48% (Figure 6E), whereas the active paxillin decreased to 60% (Figure 6D) and the active FAK to 59% (Figure 6F). In support of these results, the treatment of FX1 showed a clear reduction of these proteins and their phosphorylated form (Figure 6C–F). The same experiments were also performed with HIPEC and SGHPL-4 cells and comparable results were obtained (Figures S4 and S5). These results highlight that BCL6 is required for the recruitment and activation of the important FA proteins paxillin and FAK, and that its inhibition or depletion impairs FA assembly.

### 2.6. Knockdown of BCL6 Interferes with the Expression of Pro-Migratory Genes

**Figure 7:**
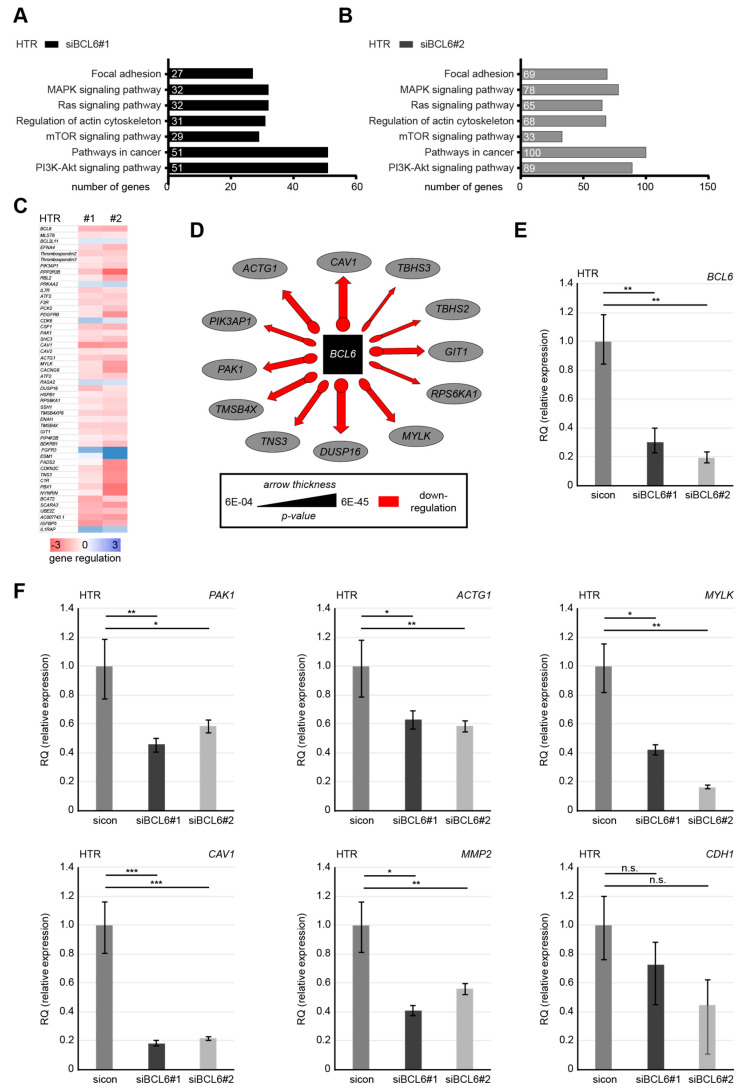
Reduction of BCL6 reduces the levels of multiple genes related to migratory machinery. (**A** and **B**) HTR cells were treated with sicon, siBCL6#1, or siBCL6#2 for 48 h and the RNA from three independent experiments was extracted for transcriptome analysis. The effects of siBCL6#1 (**A**) or siBCL6#2 (**B**) on different pathways were determined by gene ontology enrichment analysis using KEGG pathway enrichment analysis. (**C**) A heatmap of the most differently expressed genes. Gene expression was analyzed using the DESeq2 R package. Genes with a *p*-value < 0.01 and a fold change greater than 3 (blue color code) and below 3 (red color code), respectively, are included. (**D**) The most significant genes affected by the depletion of BCL6 in the context of migration are depicted. (**E**) BCL6 gene analysis as the transfection efficiency control. (**F**) The gene levels of p21 RAC1 activated kinase 1 (PAK1), actin gamma (ACTG1), myosin light chain kinase (MYLK), caveolin 1 (CAV1), and matrix metalloproteinase-2 (MMP2) were measured. The mRNA data are based on three independent experiments and presented as the RQ, with the minimum and maximum range. RQ: relative quantification of gene expression. * *p* < 0.05, ** *p* < 0.01, and *** *p* < 0.001.

Being a transcriptional modulator, BCL6 works by binding to target genes, recruiting several different chromatin-modifying co-repressor complexes, and influencing the expression of hundreds of genes [48]. To more deeply investigate the possible mechanisms by which BCL6 suppression compromises cell motility, we performed transcriptome-wide profiling with a cBot Cluster Generation System using PE Cluster Kit cBot-HS (Illumina). The data were analyzed using the DESeq2 R package for differential expression analysis and GOSeq R package for enrichment analysis (Novogene Co., Ltd., Beijing, China). Compared to HTR cells treated with control siRNA, we analyzed differently expressed genes in HTR cells depleted of BCL6. The various pathways affected by BCL6 knockdown with siBCL6#1 or siBCL6#2 were evaluated using gene ontology enrichment analysis [49]. The genes with a *p*-value smaller than 0.05 were identified, and these are related to various regulatory pathways, including FA, the actin cytoskeleton, and PI3K signaling in HTR cells deficient of BCL6 with siBCL6#1 (Figure 7A) or siBCL6#2 (Figure 7B). Genes with a *p*-value smaller than 0.01 and a fold change greater than 3 (blue color code) and below 3 (red color code), respectively, are depicted in a heatmap (Figure 7C). In particular, the knockdown of BCL6 decreased the levels of numerous genes tightly associated with the actin cytoskeleton and cell motility (Figure 7D). Further gene analysis underlined the reduced levels of multiple genes seen by the transcriptomic data and previous work (Figure 7F), such as p21 RAC1 activated kinase 1 (*PAK1*), actin gamma (*ACTG1*), myosin light chain kinase (*MYLK*), caveolin 1 (*CAV1*), and matrix metalloproteinase-2 (*MMP2*), in HTR cells depleted of BCL6 (Figure 7E). Comparable results were also observed for the genes (ACTG1, CAV1, and PAK1) with HIPEC cells depleted of BCL6 using siBCL6#1 or siBCL6#2 (Figure S6A,B). Unfortunately, the gene levels of epithelial cadherin (CDH1) and MYLK were not detectable in HIPEC cells. These results suggest that BCL6 is involved in the regulation of different gene patterns related to the migratory machinery and that interfering with BCL6 compromises the migration and invasion of trophoblastic cells.

## 3. Discussion

The transcriptional repressor and proto-oncogene BCL6 mediates various crucial cellular processes by affecting a great body of diverse genes [4,48]. Intriguingly, the deregulated expression of BCL6 is associated with an increased malignancy of multiple cancer entities, such as lymphomas, breast cancer, and ovarian cancer, which correlates with increased invasion and metastatic rates [1,2,5,7,48]. Interestingly, multiple studies have reported an increased mRNA expression of BCL6 in placentas from PE patients [8,9,12], which also show impaired EVT invasion. Prior to this investigation, we demonstrated BCL6′s importance in the proliferation and survival of trophoblastic cells [13,14] and in the fusion and syncytialization of villous cytotrophoblasts [15]. Here, we show the requirement of BCL6 in the motility, migration, and invasion of first trimester trophoblastic HTR-8/SVneo, HIPEC-65, and SGHPL-4 cells through multiple mechanisms.

Cell adhesion to ECM is essential for a multicellular existence [50]. The main cellular adhesion receptors, which are integrins and FAs, are the key components of the cell migration machinery [50,51]. Interestingly, the depletion of BCL6 compromises cell adhesion accompanied by reduced integrin activation and delayed FA formation indicated by reduced levels of the adaptor protein paxillin. As BCL6 silences hundreds of genes related to diverse cellular activities [48], we assumed that some of its target genes could be involved in these events. In support of this assumption, our transcriptome analysis reveals that multiple important genes responsible for cell adhesion formation and its turnover, such as *thrombospondin 2* and *3*, which are mediators of cell adhesion and extracellular matrix remodeling [52]; *MMP2*, which is a crucial player in the breakdown of the ECM [53]; and *CAV1*, which is a promoter of cell adhesion [54], are downregulated in HTR cells depleted of BCL6, suggesting that these genes are regulated by BCL6, either directly or indirectly. The downregulation of BCL6 reduces the expression of these important modulators for cell adhesion and its dynamic regulation, compromising the first steps of the migration of trophoblastic cells. In line with compromised trophoblast migration, most of these genes are also known to be deregulated in the placentas of preeclamptic patients, as well as cancer cells, affecting their growth, invasion, and migration, further fueling the progression of PE [55,56,57] and tumor metastasis [58,59].

The interaction of adhesion receptors such as integrins with the ECM transduces environmental signals into the cellular apparatuses, such as the actin cytoskeleton, which is important for maintaining the cell shape, contractility, tension, and motility. The dynamic actin cytoskeleton provides both the protrusive and contractile forces required for cell migration via a combination of actin polymerization and depolymerization, actin filament crosslinking, and the interaction of myosin-based motors with actin filaments [50]. Intriguingly, the knockdown of BCL6 stabilizes actin filaments and reduces the dynamics of the actin cytoskeleton. Importantly, the suppression of BCL6 decreases the gene level of *PAK1*, which is an important serine/threonine kinase crucial in actin and microtubule dynamics, by phosphorylating various substrates such as paxillin [60], stathmin [61], vimentin [62], and filamin [63]. In fact, reduced BCL6 is accompanied by less phosphorylated paxillin. Moreover, reduced BCL6 is associated with decreased gene levels of numerous critical regulators responsible for the assembly and dynamics of the actin cytoskeleton, including *ACTG1* encoding gamma actin, which is one of the main components of the actin cytoskeleton and a mediator of cell migration [64]; *MYLK*, which is a kinase phosphorylating myosin regulatory light chains to facilitate myosin interaction with actin filaments [65]; *TMSB4X*, the gene for thymosin-β4 and an actin sequester important in actin polymerization [66]; and protein phosphatase Slingshot homolog 1 (*SSH1*), which is a player in actin dynamics that reactivates cofilin [67]. These data highlight that BCL6 is a critical regulator for the regulation of the actin cytoskeleton and its deregulation hampers the dynamics of the actin cytoskeleton, impairing the motility of trophoblastic cells.

Furthermore, integrin adhesion activates FAK, MAPK/Erk, and PI3K/Akt signaling [51]. Actually, the depletion of BCL6 reduces the protein signals of *p*-FAK, *p*-Akt, and *p*-Erk1/2 and is associated with a decreased migratory and enhanced stationary cell type. In support of this notion, reduced BCL6 leads to altered gene levels of important regulators for MAPK/Erk and PI3K/Akt, such as *CAV1* [54], ribosomal protein S6 kinase alpha-1 (*RPS6KA*) [68], and dual specificity protein phosphatase 16 (*DUSP16*) [69]. Multiple signaling pathways, including MAPK/Erk, PI3K/Akt, and FAK signaling, have been reported to be involved in controlling the migration and invasion of trophoblastic cells [70,71]. Additionally, FAK phosphorylation at Y397 is crucial for cell invasion and the activity of MMP2 [72]. Here, we show that the depletion of BCL6 inhibits the activation of FAK with a reduced level of *MMP2*. The inhibition of these multiple stated upstream kinases reduces cell adhesion and deregulates actin dynamics, hampering the recruitment and activation of FA components such as paxillin and FAK. This deregulation contributes to decreased cell migration and invasion in cancer and trophoblastic cells.

## 4. Materials and Methods

### 4.1. Cell Culture, siRNA Transfection, Plasmid Cloning, Transfection, and Western Blot

BeWo (Sigma-Aldrich, Taufkirchen), HTR-8/SVneo (HTR) [29], HIPEC-65 [30], and SGHPL-4 cells [37] were cultured as instructed. siRNA targeting BCL6#1 (sense: CCUUGUGACAAGGCCAGCA and antisense: UGCUGGCCUUGUCACAAGG) was manufactured by Sigma-Aldrich. Control siRNA and the second siRNA targeting BCL6 (siBCL6#2) (“cocktail” consisting of several siRNAs targeting the coding region) were obtained from Qiagen (Hilden). Cells were transiently transfected using Oligofectamine^TM^ (Thermo Fisher Scientific), as previously reported [14,15,74]. For the cloning of BCL6 constructs, the cDNA of full-length human BCL6 was obtained from RZPD (Berlin) and cloned into Kpn1/BamH1 sites (fw: 5′-GTA CGG TAC CGA TGG CCT CGC CGG CTG ACA GCT GTA TCC, rev: 5′-GAT CGG ATC CTC AGC AGG CTT TGG GGA GCT CCG) of p3xFLAG-CMV-10 (Invitrogen, Carlsbad, Germany).

Western blot analysis was performed as previously described [35,75]. The following antibodies were used for Western blot visualization: Mouse monoclonal antibody against BCL6 (DAKO, Hamburg, Germany); monoclonal antibodies against β-actin and FLAG^®^ M2 (Sigma-Aldrich, St. Louis, MI, USA); rabbit polyclonal antibody against FAK (Cell Signaling Frankfurt, Germany, #3285S); rabbit monoclonal antibody against *p*-FAK (Y397) (Cell Signaling, #8556S); rabbit polyclonal antibody against AKT (Cell Signaling, #9272S); rabbit polyclonal antibody against *p*-AKT (S473) (Cell Signaling, #9271S); rabbit polyclonal antibody against ERK1/2 (Merck Millipore, Darmstadt, #16-111); rabbit monoclonal antibody against *p*-ERK1/2 (T202/Y204) (Cell Signaling, #9101); rabbit monoclonal antibody against STAT3 (Cell Signaling, #30835); and rabbit monoclonal antibody against *p*-STAT3 (Y705) (Cell Signaling, #9145).

### 4.2. Indirect Immunofluorescence Staining, Immunohistochemistry of Placental Tissue, and Intensity Measurement

Indirect immunofluorescence staining was performed as previously described [74,76,77]. The following primary antibodies were used for staining: Rat monoclonal antibodies against active β1-integrin (CD29, clone 9EG7) (BD Biosciences, Heidelberg, Germany); rabbit polyclonal antibody against FAK (Cell Signaling, #3285S); rabbit monoclonal antibody against *p*-FAK (Y397) (Cell Signaling, #8556S); monoclonal mouse antibody against paxillin (BD Biosciences, Heidelberg, Germany, #610052); and rabbit monoclonal antibody against *p*-paxillin (Y118) (#2541S) and mouse monoclonal antibody against α-tubulin (Sigma-Aldrich, St. Louis, MI, USA, #F2168). The F-actin cytoskeleton was stained using phalloidin (Phalloidin-Atto 550; Sigma-Aldrich, St. Louis, MI, USA). FITC-, Cy3-, and Cy5-conjugated secondary antibodies were obtained from Jackson Immunoresearch (Cambridgeshire). DNA was stained using 4′,6-diamidino-2-phenylindole-dihydrochlorid (DAPI) (Roche). Imaging of the immunofluorescence slides was performed with an Axio Imager 7.1 microscope (Carl Zeiss, Hallbergmoos) or by confocal laser scanning microscopy (CLSM, Leica CTR 6500, Heidelberg, Germany), as described previously [74,76,77].

A standard staining procedure with DAKO EnVision^TM^ FLEX peroxidase blocking reagent (K8000, DAKO, Hamburg, Germany) was used to stain formalin-fixed, paraffin-embedded first trimester placental tissue sections from healthy individuals, as previously described [78]. A mouse monoclonal antibody against BCL6 (DAKO, Hamburg, Germany) was used for staining. All slides were counterstained with hematoxylin and analyzed using an AxioObserver.Z1 microscope (Zeiss, Göttingen, Germany).

The image acquisition was performed with the same wave intensity and exposure time. Images shown in figures are maximum intensity projections of confocal Z-sections. The mean fluorescence intensities of multiple proteins (FAK, pFAK, paxillin, *p*-paxillin, and F-actin (phalloidin) were quantified by using the LAS AF Lite software from Leica. The mean signal size, mean gray intensity of paxillin, and active integrin area were analyzed with NIH ImageJ, as previously reported [42].

### 4.3. Cell Adhesion, Migration, and Invasion Assay

For cell spreading/adhesion assays, four-chamber slides were coated with 2 µg/mL fibronectin for 15 min (Merck Millipore, Darmstadt), as previously described [79]. Cells were trypsinized, reseeded on fibronectin-coated slides (HTR, 3.5 × 10^4^, HIPEC, 4.5 × 10^4^ cells per chamber), and incubated at 37 °C for 20 and 60 min. Non-adherent cells were removed by careful washes with PBS and adherent cells were counted. Adherent cells were fixed with paraformaldehyde containing Triton X-100 and stained. Quantification of the cell area from slides released at 20 and 60 min post plating was analyzed using ImageJ.

To evaluate the dynamics of the actin cytoskeleton, cells were starved in serum-free medium for 12 h. Cells were treated with latrunculin B (200 nM; BIOMOL GmbH, Hamburg) for 90 min to depolymerize actin fibers. The drug was washed out with PBS and the cells were released into serum-containing medium for 30 and 60 min. The cells were fixed with 4% paraformaldehyde containing 0.2% Triton X-100 for 15 min at room temperature before processing for immunofluorescence staining.

Cell migration assays were performed, as previously reported [80], with culture-inserts from ibidi (Martinsried). Culture-inserts (cell-free gap of 500 µm) were placed in a 6-cm culture dish and both wells of each insert were filled with cell suspension. HTR (6.5 × 10^4^) and HIPEC (5.5 × 10^4^) and BeWo cells (5.5 × 10^4^) were seeded in each well of the culture-inserts. Culture-inserts were gently removed after at least 8 h. The cells were acquired and imaged at indicated time points with bright-field images. Four pictures of each insert were taken and the experiments were performed in triplicate and with at least three independent times. The open area was measured using the AxioVision SE64 Re. 4.9 software (Zeiss).

For the invasion assay, HTR cells depleted of BCL6 or control HTR cells were used as previously described [80]. Individual invaded cells were quantified with a fluorescence microscope.

### 4.4. Cell Motility Assay

The motility assay was performed with an AxioObserver.Z1 microscope (Zeiss), imaged with an AxioCam MRc camera (Zeiss) equipped with an environmental chamber to maintain proper environmental conditions (37 °C, 5% CO2). Time-lapse movies of 13 h were analyzed and quantified by using ImageJ 1.49i software (National Institutes of Health, USA) with the manual tracking plugin, and Chemotaxis and Migration Tool (Ibidi GmbH, Germany), as described previously [33].

### 4.5. RNA Extraction, Real-Time PCR, and Transcriptomic Analysis

Total RNAs were extracted with the EXTRACTME Total RNA Kit without DNase digestion, according to manual instructions (7Bioscience GmbH, Hartheim). Reverse transcription was performed using Go Script Reverse Transcription Mix, as instructed (Promega, Mannheim). The primers and probes for *GAPDH* (Hs_02786624), *BCL6* (Hs00153368_m1), *PAK1* (Hs00945621_m1), *ACTG1* (Hs03044422_g1), *MYLK* (Hs00364926_m1), *CAV1* (Hs00971716_m1), *MMP2* (Hs01548727_m1), and *CDH1* (Hs01023895_m1) were obtained from Applied Biosystems (Darmstadt). Real-time PCR was performed with a StepOnePlus Real-time PCR System and the data were analyzed via StepOne Software v2.3 (Applied Biosystems). The final results are presented as relative quantification (RQ), as previously described [81,82].

For transcriptomic analysis, the HTR cell line was treated with siRNA targeting BCL6#1 and #2 or control siRNA for 48 h. The experiment was repeated three independent times and total RNAs were extracted with the EXTRACTME Total RNA Kit, according to manual instructions (7Bioscience GmbH, Hartheim, Germany) and without DNase digestion. A total amount of 1 μg RNA per sample was sent to Novogene Co., Ltd. (Beijing, China) and used as input material for the RNA sample preparations. Sequencing libraries were generated using the NEBNext^®^ Ultra^TM^ RNA Library Prep Kit for Illumina^®^ (NEB, USA), following the manufacturer’s recommendations. Differential expression analysis between two conditions/groups (three biological replicates per condition) was performed using the DESeq2 R–package and 60,450 genes were analyzed. The genes found by DESeq2 were further depicted in different pathways by GO enrichment analysis and KEGG pathway enrichment analysis.

### 4.6. Statistical Analysis

To evaluate the significance of the cell viability and invasion assay, Student’s *t*-test (two-tailed and paired or homoscedastic) was used. An unpaired Mann–Whitney U test (two-tailed) was used to analyze the statistical significance of the single cell tracking and fluorescence intensity quantification for the proteins (FAK, pFAK, *p*-paxillin, active integrin, and actin fibers (phalloidin)). Differences were considered statistically significant when *p* < 0.05. For transcriptomic analysis, a differential expression analysis of two conditions was performed using the DEGseq R package. The *p* values were adjusted using the Benjamini and

Hochberg methods. A corrected p-value of 0.005 and |log2^(Fold Change)^| of 1 were set as the threshold for significantly differential expression. Student’s t-test (two-tailed and paired or homoscedastic) was used to evaluate the significant difference between diverse groups with real-time PCR.

## 5. Conclusions

Taken together, in combination with our previous results [13,14,15], we have demonstrated that BCL6 is crucial for various functions of trophoblasts, including proliferation, survival, fusion, migration, and invasion. As BCL6 is strongly expressed in EVTs of the proximal cell columns of first trimester placentas and SGHPL-4, HIPEC-65, and HTR-8/SVneo cells are all derived from first trimester placentas [29,30,37], these observations suggest an important role of BCL6 in early stages of EVT invasion into the maternal decidua. Further investigations are needed to delineate the exact working mechanisms by which deregulated BCL6 contributes to the development of preeclampsia.

Given that BCL6 is an important oncogene involved in diverse malignant entities, studies on its presence in trophoblasts could provide novel insights into common signaling pathways shared by malignant progression and placental development, as suggested by multiple deregulated signaling pathways related to cell motility and migration [18]. Indeed, the genes *ACTG1*, *CAV1*, *MYLK*, and *PAK* were also found to be deregulated in a transcriptomic analysis of epidermoid carcinoma cells A431 [73], indicating that BCL6 modulates the invasion capacity of physiological and malignant components through similar molecular mechanisms.

## Supplementary Materials

The following are available online at https://www.mdpi.com/1422-0067/21/21/8393/s1: Figure S1. Inhibition of BCL6 reduces the migration of trophoblastic cells; Figure S2. Suppression of BCL6 reduces the motility of first trimester trophoblastic cells; Figure S3. Cell adhesion and integrin activation are compromised in HIPEC cells with reduced BCL6; Figure S4. Reduced paxillin, p-paxillin, FAK, and p-FAK at FAs of HIPEC cells deficient of BCL6; Figure S5. Reduced paxillin, p-paxillin, FAK, and p-FAK at FAs of SGHPL-4 cells deficient of BCL6; Figure S6. Reduction of BCL6 reduces the levels of ACTG1, PAK1, and CAV1 related to migratory machinery in HPEC cells.

## Abbreviations

PE	Preeclampsia
BCL6	B-cell lymphoma
siRNA	small interfering RNA
FA	Focal adhesion
FAK	Focal adhesion kinase
vCTB	villous trophoblastic cells
EVTs	extravillous trophoblasts
STBs	syncytiotrophoblasts
HIPEC	HIPEC-65
HTR	HTR-8/SVneo
PI3K	phosphoinositide 3-kinase
Akt	Proteinkinasen B (PKBα/β/γ)
Erk1/2	extracellular regulated kinase 1/2
MAPK	mitogen-activated protein kinase
STAT3	signal transducer and activator of transcription 3
PAK1	p21 RAC1 activated kinase 1
ACTG1	actin gamma
MYLK	myosin light chain kinase
CAV1	caveolin 1
MMP2	matrix metalloproteinase-2
CDH1	epithelial cadherin
SSH1	protein phosphatase Slingshot homolog 1
RPS6KA	ribosomal protein S6 kinase alpha-1
DUSP16	dual specificity protein phosphatase 16
