# Supplementary material for: The Function of Oncogene B-Cell Lymphoma 6 in the Regulation of the Migration and Invasion of Trophoblastic Cells
Source: Int J Mol Sci. 2020 Nov 9;21(21):8393. doi: 10.3390/ijms21218393 (PMC7664908; doi:10.3390/ijms21218393)

Supplementary Information

Figure. S1 Inhibition of BCL6 reduces migration of trophoblastic cells.
(A and B) HTR (A) and BeWo (B) cells were treated with BCL6 inhibitor 79-6 with indicated concentrations (25 μM, 50 μM, 100 μM) for indicated time periods (A: 0 h, 12 h, and 24 h; B: 0 h, 24 h, 48 h, and 72 h). Treated cells were subjected to wound healing/migration assays and images were taken to document the migration front. The open areas between both migration fronts were quantified at various time points. The cell-free area at 0 h was assigned as 100%. The results from three independent experiments are presented as mean ± SEM. $^{**}p < 0.01$, $^{*}p < 0.05$. (C-E) BeWo cells were depleted of BCL6 (D) and subjected to migration assay. Images were taken to document the migration front (C) and the open areas between both migration fronts were quantified at various time points (E). The cell-free area at 0 h was assigned as 100%. The results from three independent experiments are presented as mean ± SEM. $^{***}p < 0.001$.

Fig. S1

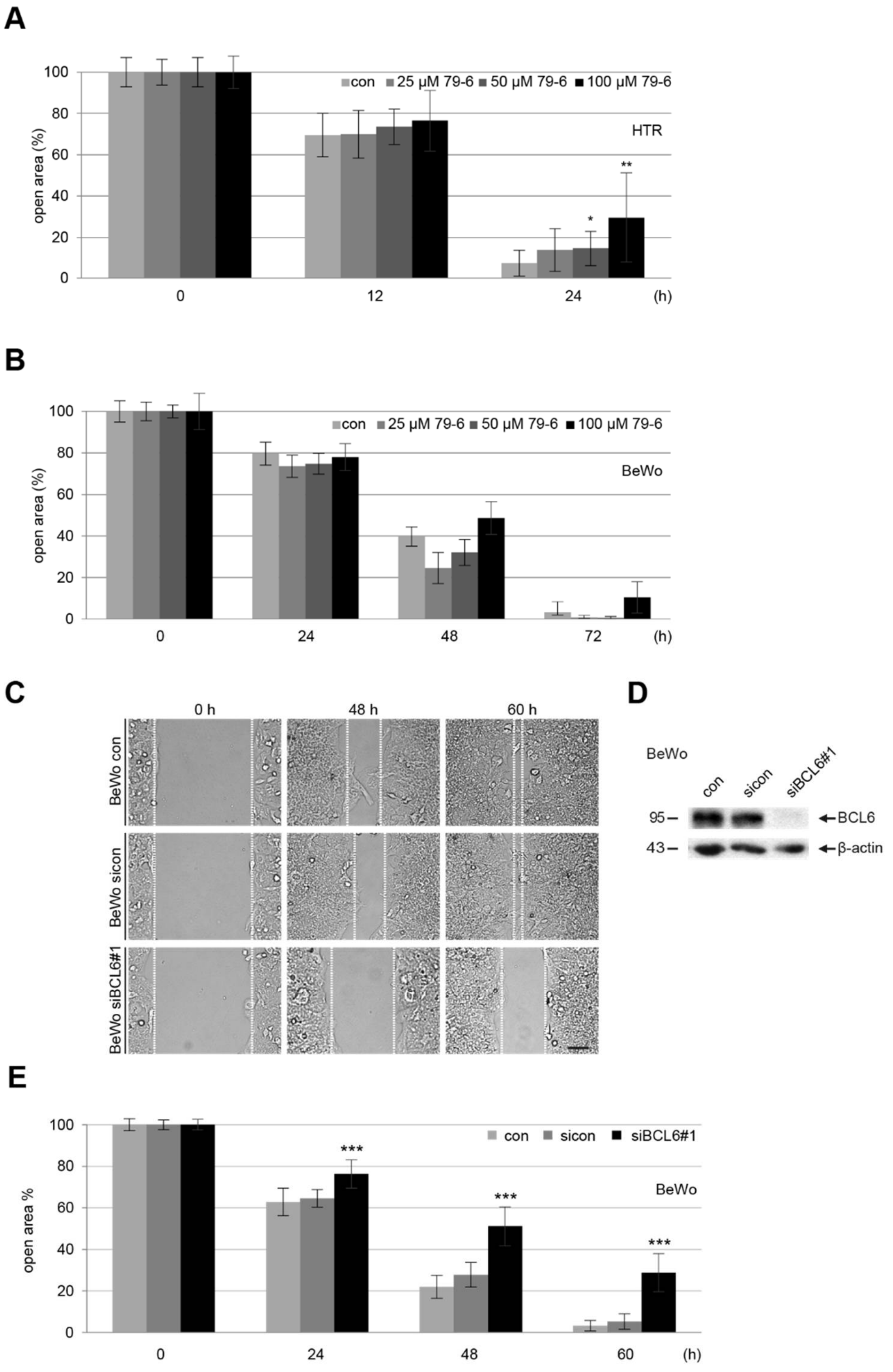
A
open area (%)
con
25 µM 79-6
50 µM 79-6
100 µM 79-6
HTR
(h)
B
open area (%)
BeWo
(h)
C
0 h
48 h
60 h
BeWo con
BeWo sicon
BeWo siBCL6#1
D
BeWo
con
sicon
siBCL6#1
95
43
BCL6
β-actin
E
open area %
con
sicon
siBCL6#1
BeWo
(h)

Figure. S2 Suppression of BCL6 reduces motility of 1st trimester trophoblastic cells.

(A and B) Time-lapse microscopy was performed with HIPEC (A) and SGHPL-4 (B) cells, which were depleted of BCL6 with siBCL6#1 or treated with the BCL6 inhibitor FX1 for up to 12 h. Random motility of these cells was analyzed. Representative trajectories of individual cells (n=30) are shown. In HIPEC cells evaluated accumulated distance, velocity and directionality are depicted in Fig. 2E-G. (C-F) Evaluated accumulated distance (C), velocity (D) and directionality (E) from three independent experiments are shown as scatter plots with variations. ***$p < 0.001$, *$p < 0.05$. Relative gene levels of BCL6 were evaluated in treated SGHPL-4 cells as transfection efficiency control (F). (G-K) HTR cells were transfected with Flag-BCL6 plasmids and subjected to time-lapse microscopy. Random motility of these cells was analyzed. Representative trajectories of individual cells (n=30) are shown (G). Western blot analysis serves as transfection efficiency control (H). Evaluated accumulated distance (I), velocity (J) and directionality (K) from three independent experiments are shown as scatter plots with variations. **$p < 0.01$.

Fig. S2

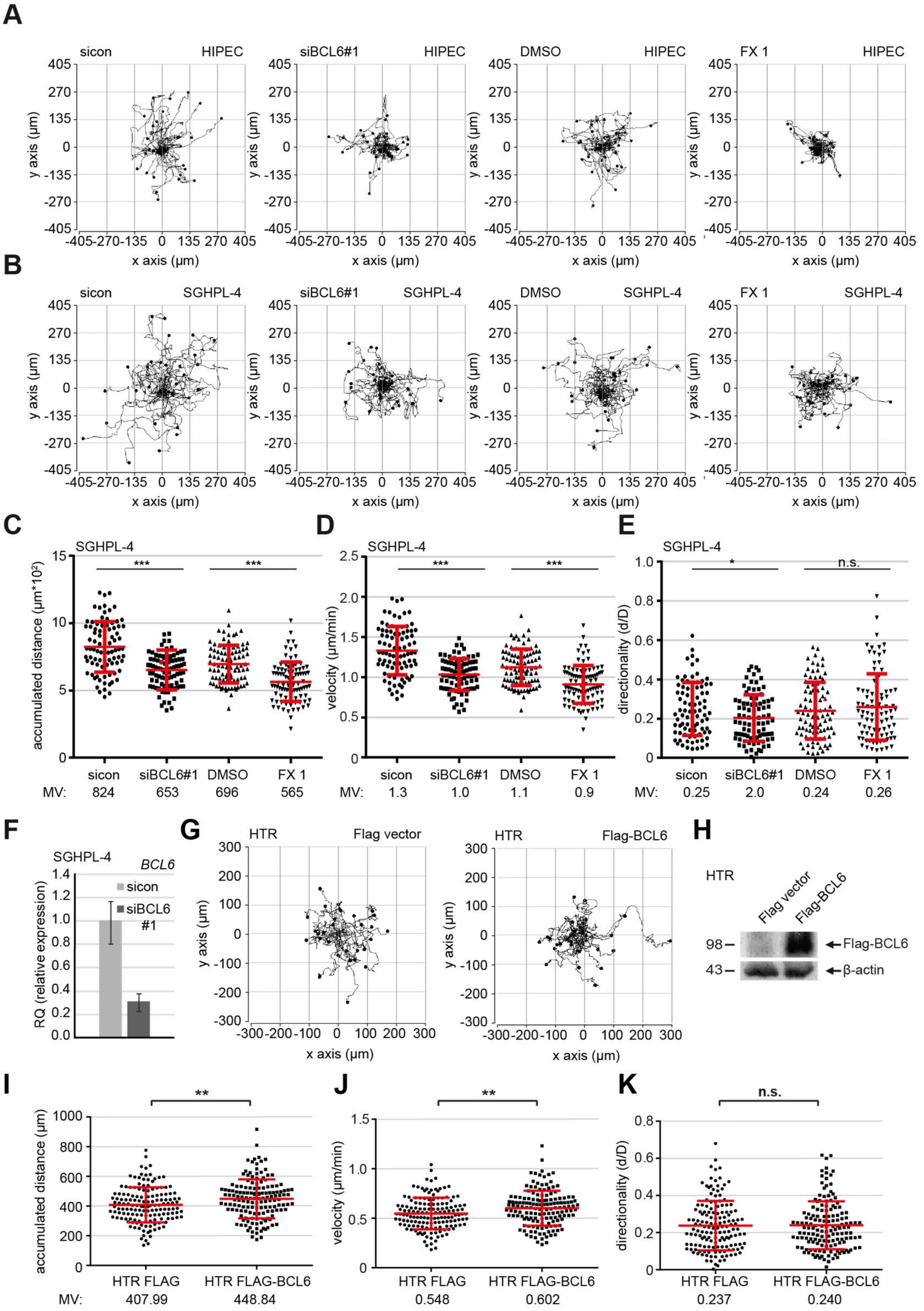
A
sicon HIPEC
siBCL6#1 HIPEC
DMSO HIPEC
FX 1 HIPEC
y axis (µm)
x axis (µm)
B
sicon SGHPL-4
siBCL6#1 SGHPL-4
DMSO SGHPL-4
FX 1 SGHPL-4
C
SGHPL-4
accumulated distance (µm*10²)
sicon siBCL6#1 DMSO FX 1
MV: 824 653 696 565
D
SGHPL-4
velocity (µm/min)
sicon siBCL6#1 DMSO FX 1
MV: 1.3 1.0 1.1 0.9
E
SGHPL-4
directionality (d/D)
n.s.
sicon siBCL6#1 DMSO FX 1
MV: 0.25 2.0 0.24 0.26
F
SGHPL-4
BCL6
sicon
siBCL6 #1
RQ (relative expression)
G
HTR Flag vector
HTR Flag-BCL6
H
HTR
Flag vector
Flag-BCL6
98
43
Flag-BCL6
β-actin
I
accumulated distance (µm)
HTR FLAG HTR FLAG-BCL6
MV: 407.99 448.84
J
velocity (µm/min)
HTR FLAG HTR FLAG-BCL6
0.548 0.602
K
n.s.
directionality (d/D)
HTR FLAG HTR FLAG-BCL6
0.237 0.240

Figyre. S3 Cell adhesion and integrin activation are compromised in HIPEC cells with reduced BCL6.

(A-C) HIPEC cells were treated with sicon, siBCL6#1 or siBCL6#2. 48 h later treated cells were trypsinized and reseeded for 20 or 60 min for further evaluation of attached cells or immunofluorescence staining. Attached cells were quantified at 20 min (A) and at 60 min (B) after reseeding (300 cells for each condition). The results are based on three independent experiments and presented as box plots. The BCL6 gene levels were measured in treated cells as transfection efficiency controls (C). (D and E) Attached cells at 20 min (C) and 60 min (D) after reseeding were also stained for the focal adhesion (FA) markers paxillin and active integrin, and DNA for evaluation. Relative intensity of paxillin was evaluated at 20 min (D) and 60 min after reseeding (E) (300 FAs for each condition). The data are from three independent experiments and presented as scatter plots with variations. (F and G) Relative intensity of active integrin was evaluated at 20 min (F) and 60 min after reseeding (G) (300 FAs for each condition). The results are from three independent experiments and presented as scatter plots with variations. (H and I) Relative cell size was evaluated at 20 min (H) and 60 min after reseeding (I) (30 cells for each condition). The data are from three independent experiments and presented as scatter plots with variations. ***$p < 0.001$, **$p < 0.01$, *$p < 0.05$.

Fig. S3

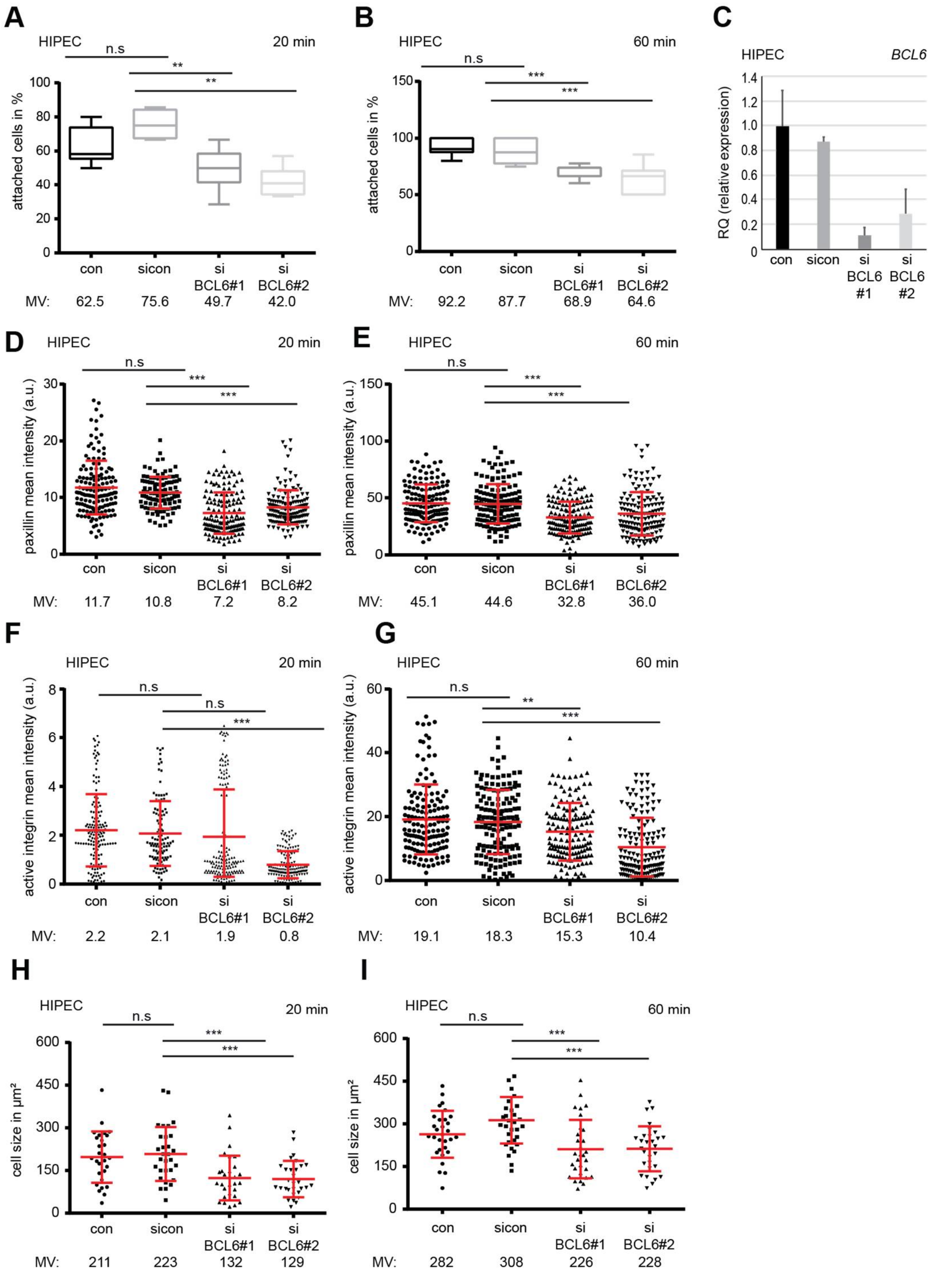
A
HIPEC
20 min
n.s
**
attached cells in %
con
sicon
si BCL6#1
si BCL6#2
MV: 62.5 75.6 49.7 42.0
B
HIPEC
60 min
***
MV: 92.2 87.7 68.9 64.6
C
HIPEC
BCL6
RQ (relative expression)
si BCL6 #1
si BCL6 #2
D
paxillin mean intensity (a.u.)
MV: 11.7 10.8 7.2 8.2
E
MV: 45.1 44.6 32.8 36.0
F
active integrin mean intensity (a.u.)
MV: 2.2 2.1 1.9 0.8
G
MV: 19.1 18.3 15.3 10.4
H
cell size in µm²
MV: 211 223 132 129
I
MV: 282 308 226 228

Figure. S4 Reduced paxillin, p-paxillin, FAK and p-FAK at FAs of HIPEC cells deficient of BCL6. (A and B) HIPEC cells were treated with sicon, siBCL6#1, DMSO or the BCL6 inhibitor FX1. 24 h later cells were stained for paxillin (green), p-paxillin (red) and DNA (blue, DAPI) (A), or for FAK (green), p-FAK (red) and DNA (blue, DAPI) (B). Scale: 50 μm. Insets are magnified regions. Scale: 25 μm. (C and D) Mean intensities of paxillin (C) and of p-paxillin (D) were measured (300 FAs for each condition). The results are derived from three independent experiments and presented as scatter plots with variations. a.u., arbitrary units. (E and F) Mean intensities of FAK (E) and of p-FAK (F) were measured (300 FAs for each condition). The results are from three independent experiments and presented as scatter plots with variations. a.u., arbitrary units. (G and H) Mean size of paxillin (G) or FAK (H) in FAs was measured (300 FAs for each condition). The results are from three independent experiments and presented as scatter plots with variations. For C-H: ***$p < 0.001$, **$p < 0.01$. (I) The BCL6 gene levels were measured in HIPEC cells as transfection efficiency controls.

Fig. S4

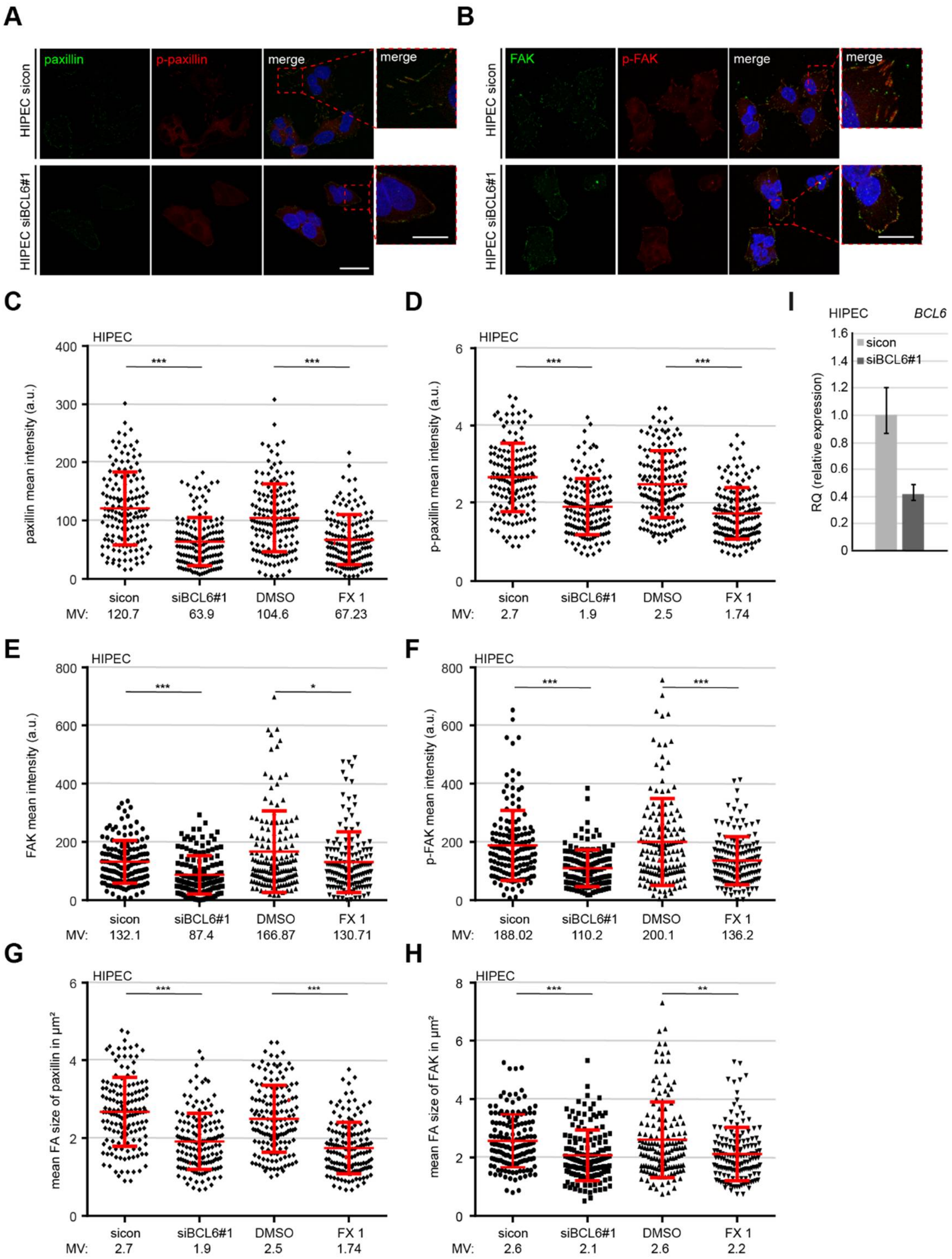
A
paxillin
p-paxillin
merge
HIPEC sicon
HIPEC siBCL6#1
B
FAK
p-FAK
merge
C
HIPEC
paxillin mean intensity (a.u.)
sicon
siBCL6#1
DMSO
FX 1
MV: 120.7 63.9 104.6 67.23
D
p-paxillin mean intensity (a.u.)
MV: 2.7 1.9 2.5 1.74
I
HIPEC
BCL6
RQ (relative expression)
E
FAK mean intensity (a.u.)
MV: 132.1 87.4 166.87 130.71
F
p-FAK mean intensity (a.u.)
MV: 188.02 110.2 200.1 136.2
G
mean FA size of paxillin in µm²
MV: 2.7 1.9 2.5 1.74
H
mean FA size of FAK in µm²
MV: 2.6 2.1 2.6 2.2

Figure. S5 Reduced paxillin, p-paxillin, FAK and p-FAK at FAs of SGHPL-4 cells deficient of BCL6. (A and B) SGHPL-4 cells were treated with sicon, siBCL6#1, DMSO or the BCL6 inhibitor FX1. 24 h later cells were stained for paxillin, p-paxillin and DNA, or for FAK, p-FAK and DNA. Mean intensities of paxillin (A) and of p-paxillin (B) were measured (150 FAs for each condition). The results are derived from three independent experiments and presented as scatter plots with variations. a.u., arbitrary units. (C and D) Mean intensities of FAK (C) and of p-FAK (D) were measured (150 FAs for each condition). The results are from three independent experiments and presented as scatter plots with variations. a.u., arbitrary units. (E and F) Mean size of paxillin (E) or FAK (F) in FAs was measured (150 FAs for each condition). The results are from three independent experiments and presented as scatter plots with variations. For C-H: ***$p < 0.001$, **$p < 0.01$. (G) The gene levels of BCL6 in treated cells were measured as transfection efficiency controls.

Fig. S5

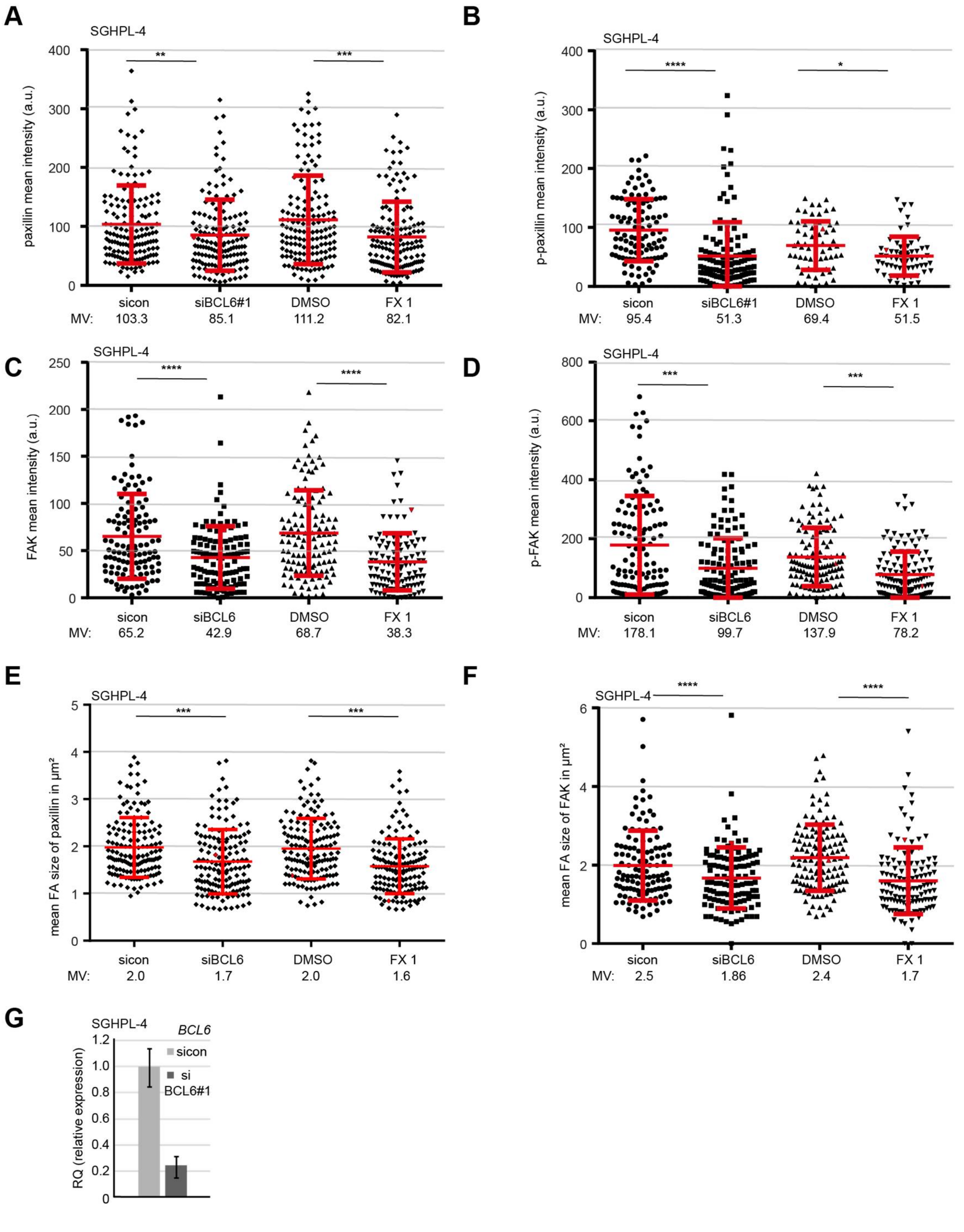
A
SGHPL-4
paxillin mean intensity (a.u.)
400
300
200
100
0
**
***
sicon
siBCL6#1
DMSO
FX 1
MV: 103.3 85.1 111.2 82.1
B
SGHPL-4
p-paxillin mean intensity (a.u.)
****
*
sicon
siBCL6#1
DMSO
FX 1
MV: 95.4 51.3 69.4 51.5
C
SGHPL-4
FAK mean intensity (a.u.)
250
200
150
100
50
0
****
****
sicon
siBCL6
DMSO
FX 1
MV: 65.2 42.9 68.7 38.3
D
SGHPL-4
p-FAK mean intensity (a.u.)
800
600
400
200
0
***
***
sicon
siBCL6
DMSO
FX 1
MV: 178.1 99.7 137.9 78.2
E
SGHPL-4
mean FA size of paxillin in µm²
5
4
3
2
1
0
***
***
sicon
siBCL6
DMSO
FX 1
MV: 2.0 1.7 2.0 1.6
F
SGHPL-4
mean FA size of FAK in µm²
6
4
2
0
****
****
sicon
siBCL6
DMSO
FX 1
MV: 2.5 1.86 2.4 1.7
G
SGHPL-4
BCL6
RQ (relative expression)
1.2
1.0
0.8
0.6
0.4
0.2
0
sicon
si BCL6#1

Figure. S6 Reduction of BCL6 reduces the levels of *ACTG1*, *PAK1*, and *CAV1* related to migratory machine in HPEC cells.

(A and B) The gene levels of *ACTG1*, *PAK1*, *CAV1*, and *BCL6* (B) are measured. BCL6 gene analysis served as transfection efficiency control. The mRNA data are based on three independent experiments and presented as RQ with minimum and maximum range. RQ: relative quantification of gene expression. *p < 0.05, **p < 0.01.

Fig. S6

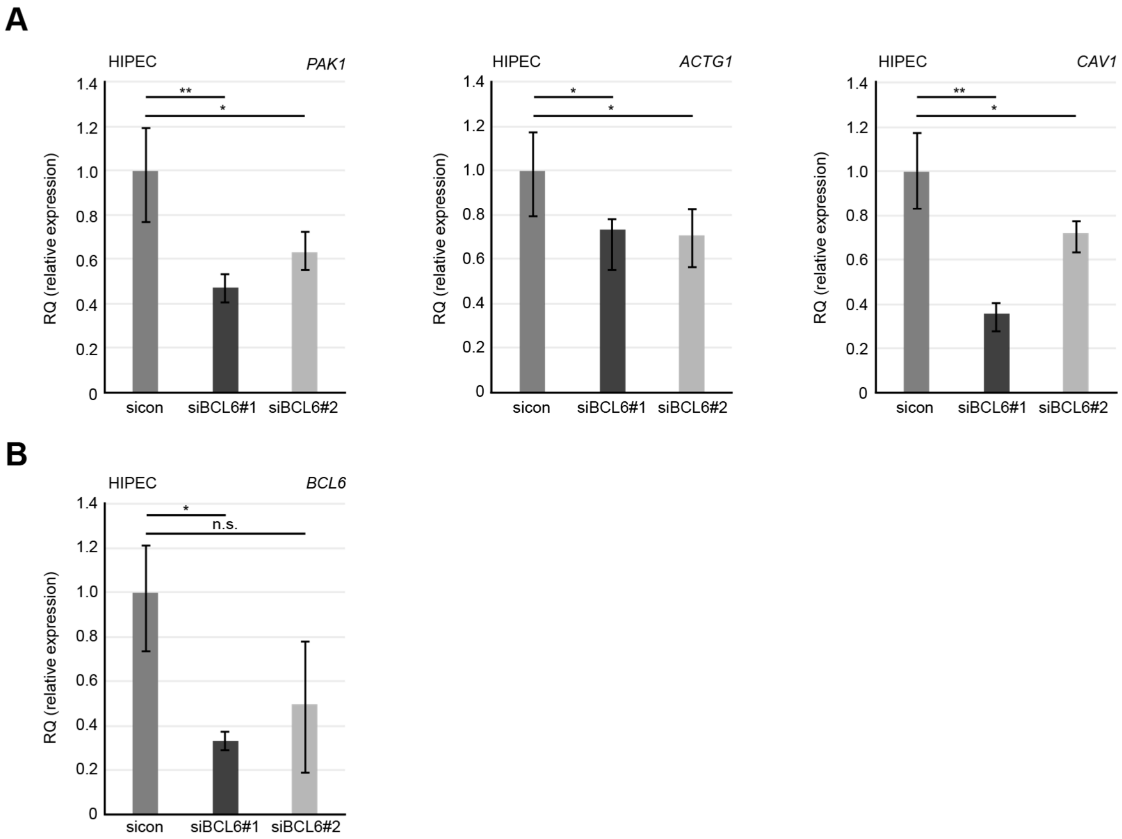

Supplement: Supplementary file 1 [file ijms-21-08393-s001.pdf]
